# Postbiotics from lactic acid bacteria as promising alternatives against urinary tract pathogens

**DOI:** 10.3389/fmicb.2026.1741001

**Published:** 2026-04-07

**Authors:** Romina Elisa D’Almeida, Mariana Grillo-Puertas, Lourdes Rocío Robles, Elvira María Hebert, Viviana Andrea Rapisarda, Josefina María Villegas

**Affiliations:** 1 Instituto Superior de Investigaciones Biológicas (INSIBIO), CONICET-UNT, Instituto de Química Biológica “Dr Bernabé Bloj”, Facultad de Bioquímica, Química y Farmacia, UNT, San Miguel de Tucumán, Argentina; 2 Centro de Referencia para Lactobacilos (CERELA-CONICET), San Miguel de Tucumán, Argentina

**Keywords:** antimicrobial activity, biofilm, *Caenorhabditis elegans*, lactobacilli, postbiotics, urinary tract infection

## Abstract

Urinary tract infections (UTIs) are one of the most common bacterial infections in hospital settings and are increasingly difficult to treat due to the rise of antibiotic-resistant uropathogens (UP). Therefore, exploring alternatives to antibiotic therapy is crucial to control the spread of these pathogens. Lactic acid bacteria (LAB) have demonstrated broad antimicrobial activity against various microorganisms, and postbiotics, bioactive compounds derived from these probiotic bacteria, are emerging as a promising tool for controlling such infections. In this study, several LAB strains exhibited antagonistic activity against different clinical UP isolates. To assess whether this activity was due to secreted metabolites, cell-free supernatants (CFSs) were obtained from 24 h LAB cultures. CFSs exhibited variable antimicrobial activity against most UP isolates. Notably, these inhibitory effects were completely lost after pH neutralization and partially reduced by heat or protease treatment, suggesting the involvement of organic acids and/or proteinaceous molecules. CFSs were able to inhibit biofilm formation and partially disrupt mature biofilms of *Staphylococcus aureus* and *Pseudomonas aeruginosa,* demonstrating their potential to manage persistent infections. Finally, safety assessment in *Caenorhabditis elegans* demonstrated that concentrations up to 25% were non-toxic, with no adverse effects on survival, locomotion, or reproduction. Using this infection model, CFS from *Lactiplantibacillus paraplantarum* CRL 1905 enhanced survival against both pathogens when administered before, during, or after infection, with dose-dependent protective effects. Our findings highlight the potential of LAB-derived postbiotics as safe and effective strategies for preventing and controlling UTIs, particularly in the context of rising antibiotic resistance.

## 1 Introduction

Urinary tract infections (UTIs) are among the most prevalent bacterial infections worldwide (Klein and Hultgren, 2020), contributing to increased mortality and morbidity rates, as well as substantial healthcare costs associated with treatment. Most of these infections are caused by diverse pathogens, such as *Escherichia coli, Klebsiella pneumoniae, Pseudomonas aeruginosa, Staphylococcus aureus, Enterococcus faecalis*, and *Proteus mirabilis* (Flores-Mireles et al., 2015; Wagenlehner et al., 2020). UTI treatments consist of long-term administration of antibiotics (ATB), often yielding suboptimal outcomes and becoming the forefront of the ATB resistance problem.

Formation of bacterial biofilm on urinary catheters is a frequent problem in UTIs. Biofilms are highly organized communities of cells attached to different biotic and abiotic surfaces that contribute to chronic human infections (Vestby et al., 2020). Uropathogens (UP) within biofilm are ~1,000-times more resistant to conventional antibiotic treatment than planktonic cells, becoming a challenge for their eradication (Pokharel et al., 2022; Shree et al., 2023).

Considered an appropriate alternative strategy to control different pathogenic bacteria, the use of lactic acid bacteria (LAB) have become a relevant research topic in recent years. Lactobacilli are Generally Recognized as Safe (GRAS) and are among the most common probiotics. Certain strains of these microorganisms exhibit a well-documented antimicrobial activity that is exerted through different mechanisms, such as nutrient competition, immune-stimulation, and production of inhibitory compounds (organic acids, ethanol, fatty acids, hydrogen peroxide, or bacteriocins) (Mokoena, 2017; Szczerbiec et al., 2022). Although many studies have demonstrated the health benefits of host-related consumption of probiotics, their clinical application in immunocompromised patients remains limited due to the risk of bacteremia caused by species such as *Lactobacillus* (Kothari et al., 2019; Kullar et al., 2023). An alternative approach involves the use of metabolic byproducts secreted by probiotic bacteria or released after bacterial lysis, known as postbiotics, which could minimize or avoid these potential adverse effects (Vinderola et al., 2022; Salminen et al., 2021). This study aimed to evaluate the antimicrobial and antibiofilm activities of postbiotics obtained from different lactobacilli strains against clinical UP, and to assess their safety and protective effects using the *Caenorhabditis elegans* infection model. The biotherapeutic potential of such probiotic-derived products is particularly relevant in the current context of increasing ATB resistance, where the emergence of multidrug-resistant pathogens highlights the urgent and critical need for alternative strategies.

## 2 Materials and methods

### 2.1 Strains and growth conditions

*Lactiplantibacillus paraplantarum* CRL 1905, *Levilactobacillus brevis* CRL 1942, *Limosilactobacillus fermentum* CRL 973, *Lactobacillus helveticus* ATCC 15009, and *Lactobacillus acidophilus* ATCC 4356 were obtained from the Culture Collection of the Centro de Referencia para Lactobacilos (CERELA-CONICET, Tucumán, Argentina) (Ruiz Rodríguez et al., 2016; Garcia-Castillo et al., 2020). LAB strains were first inoculated in MRS broth and activated twice at 37 °C for 24 h. Overnight (ON) cultures were diluted in fresh MRS broth (pH 6) to obtain an initial inoculum of 10^7^ CFU/mL, and incubated at 37 °C for 24 h under microaerophilic conditions. Then, cultures were centrifuged at 7700 × g for 10 min, and filtered through a 0.22 μm sterile filter (Millipore filter) to obtain cell-free supernatants (CFSs). Samples were stored at −20 °C until used. When mentioned, CFSs were concentrated 10 times (10x CFS) or diluted to final concentrations of 12.5, 25 and 50% (v/v).

Uropathogenic clinical isolates belong to the laboratory’s own collection (Table 1). Some were isolated from “double J” urinary catheters (Farizano et al., 2025) and others from urine cultures (UC) of adult patients, provided and previously identified by public medical centers in Tucumán. Isolates were routinely grown under aerobic conditions, in BHI medium at 37 °C with shaking (180 rpm), or in static growth at 37 °C on CLED-agar plates. Bacterial growth was monitored by measuring absorbance at 600 nm (A_600nm_) to determine turbidity in liquid medium, or by the appearance of isolated colonies in solid medium.

**Table 1:** Clinical isolates obtained from different sources.

Microorganisms	Name	Source
Gram-positive		
*Staphylococcus aureus*	Sa1	Farizano et al. (2025)
*Staphylococcus epidermidis*	Se1	Farizano et al. (2025)
	Se4	Farizano et al. (2025)
*Enterococcus faecalis*	Ef1	Farizano et al. (2025)
	Ef2	Farizano et al. (2025)
	Ef5	Farizano et al. (2025)
Gram-negative		
*Escherichia coli*	Ec1	Farizano et al. (2025)
	Ec2	Farizano et al. (2025)
	Ec3	Farizano et al., 2025
*Pseudomonas aeruginosa*	Pa1	UC (this work)
	Pa2	UC (this work)
*Klebsiella pneumoniae*	Kp1	Farizano et al. (2025)
	Kp2	Farizano et al. (2025)
	Kp4	Farizano et al. (2025)
*Proteus mirabilis*	Pm1	UC (this work)
	Pm2	UC(this work)
	Pm3	UC (this work)
*Acinetobacter baumannii*	Ab1	UC (this work)
	Ab2	UC (this work)

*Caenorhabditis elegans* N2 Bristol wild-type nematode was obtained from the Caenorhabditis Genetics Center (CGC) at the University of Minnesota (Minneapolis, MN, USA). *C. elegans* was routinely maintained on nematode growth medium (NGM) plates seeded with *E. coli* OP50 using standard procedures (Stiernagle, 2006).

### 2.2 *In vitro* antimicrobial activity assays

Two different approaches were performed to assess antimicrobial activity. Preliminary, a cross streak method was carried out using BHI plates, on which five lactobacilli strains were inoculated as 7.5-cm-long lines, and incubated at 37 °C for 24 h. Then, plates were cross-streaked with different UP isolates, and further incubated at 37 °C for 24 h. Growth inhibition near the central streak was considered a positive result.

Antimicrobial activities of CFSs obtained from the working lactobacilli strains were evaluated through the agar well diffusion assay (Balouiri et al., 2016). UP cultures, adjusted to 0.5 McFarland (~10^8^ CFU/mL), were swabbed on the surface of Mueller-Hinton agar plates, according to the standards of the Clinical and Laboratory Standards Institute (CLSI, 2021). Then, 50 μL of CFSs were added to 5 mm diameter wells. After incubation at 37 °C for 24 h, the diameter of the inhibition zone was measured.

### 2.3 Determination of minimum inhibitory concentration (MIC) and minimum bactericidal concentration (MBC)

The MIC of the tested CFSs was determined by the standard broth microdilution method (CLSI, 2021). CFSs were subjected to two-fold serial dilutions with BHI broth, ranging from 50 to 3.12%. Then, each of the diluted solutions was loaded into 96 microtiter plate wells, together with the selected UP cell suspensions. The final inoculum was prepared by adjusting ON cultures to 10^5^ CFU/mL and performing the appropriate dilutions in BHI, following CLSI recommendations. The mix was incubated for 24 h at 37 °C, and bacterial growth was determined visually. According to CLSI guidelines (CLSI, 2021), MIC was defined as the lowest concentration of CFS that resulted in complete inhibition of visible bacterial growth. MBC values were also established following CLSI criteria. Aliquots from clear wells were spread onto the surface of BHI agar plates and incubated at 37 °C for 24 h to determine viable counts. Results were expressed as CFU/mL. The MBC was set as the lowest concentration of CFS that inhibited bacterial growth by 99.9% (≥ 3 log_10_) [National Committee for Clinical Laboratory Standards (NCCLS), 1999]. All assays were performed in triplicate.

### 2.4 Cell-free supernatant characterization

To partially characterize the antimicrobial metabolite/s, each CFS (initial pH ≈ 4) was subjected to several treatments. The effect of pH and temperature on the antimicrobial activity of all CFSs was assessed by adjusting pH to 6.5 with 1 M NaOH, and by heating at boiling temperature for 30 min, respectively. Sensitivity to proteolytic activity was assayed by treating CFSs with proteinase K and trypsin (Sigma-Aldrich, Argentina), at a final concentration of 1 mg/mL for 2 h at 42 °C and 37 °C, respectively. Enzyme activities were stopped by boiling the mixture for 5 min and cooling it down to room temperature. After each treatment, the antimicrobial activity of the resulting CFSs was evaluated through the agar well diffusion assay previously described. MRS broth adjusted to pH 4 with HCl served as control. Additionally, pure lactic acid (13 mg/mL), corresponding to the concentration produced by *L. paraplantarum* CRL 1905 (Volentini et al., 2023), was tested under the same conditions as an additional control.

### 2.5 Antibiofilm activity

Biofilm formation was assayed on the basis of the ability of cells to adhere and grow on 96-well polystyrene microtiter plates (O'Toole and Kolter, 1998). ON UP cultures were diluted to an A_600nm_ = 0.1 and grown in static conditions at 30 °C in microtiter plates for 48 h. After removing the unattached cells and rinsing the plates three times with deionized water, biofilm quantification was performed using a colorimetric assay with crystal violet (CV). Briefly, 0.1% CV solution was added to each well, and the plates were incubated at room temperature for 30 min in the dark, and rinsed three times with deionized water. CV-stained attached cells were extracted with 95% ethanol. Absorbance was measured at 595 nm (SpectraMax Plus384 Absorbance Microplate Reader, US). Each experimental condition was performed using six technical replicates per assay, and the experiment was repeated three times.

The antibiofilm effect of CFSs against the selected UP strains was evaluated as described by Yang et al. (2021). Briefly, ON cultures of each UP were diluted in BHI medium to an A_600nm_ = 0.1 (~10^7^ CFU/mL), and added to 96-well plates, previously loaded with increasing dilutions of each CFS. Plates were incubated at 30 °C for 48 h. Then, biofilm quantification was determined using the crystal violet method, as described above. The percentage of biofilm formation was determined, taking as 100% the biofilm formed by the untreated UP.

In order to analyze the effect of CFSs on the eradication of preformed UP biofilms, ON cultures of UP were inoculated into 96-well plates to allow mature biofilm development, as described above. Then, planktonic cells were removed after incubation, and pre-established biofilms in each well were treated with 10x CFSs at 30 °C for 24 h. After incubation, plates were washed, and attached cells were stained with 0.1% CV. The remaining biofilm percentage was determined, taking as 100% the biofilm formed by each untreated UP. Six replicates were performed for each UP. All experiments were conducted in triplicate.

### 2.6 Safety evaluation of CFS in *Caenorhabditis elegans*

To evaluate the safety of CFS from *L. paraplantarum* CRL 1905, the small, free-living nematode *C. elegans* was employed as an *in vivo* toxicity model (Hunt, 2017). Three endpoint approaches were carried out: viability after acute and chronic CFS exposition, locomotor activity, and reproduction. All experiments were performed in triplicate, using eight replicates for each condition.

#### 2.6.1 Viability

To evaluate the potential acute and/or chronic effects of the postbiotic on viability, *C. elegans* was exposed to CFS from 1 to 10 days. Briefly, bleach-synchronized nematodes were grown on NGM plates seeded with *E. coli* OP50 until reaching the L4 larval stage (Stiernagle, 2006). Worms were then harvested using M9 buffer and washed three times by centrifugation (750 × g, 1 min) to remove residual bacteria. A total of 60–80 L4-stage nematodes were transferred to each well of a 96-well plate containing S medium (S Basal, 1 M potassium citrate, trace metals solution, 1 M CaCl₂, 1 M MgSO₄, pH 6), and CFS at concentrations of 12.5, 25 and 50% (v/v). MRS in the same percentages were used as control. Heat-killed *E. coli* OP50 resuspended in S basal (A_600nm_ = 1) was used as *C. elegans* food source, to prevent any antimicrobial effect of CFS on live bacteria or potential interactions between them. 50 μM floxuridine (FUdR) was added to prevent progeny, without affecting adult physiology or survival in our 10-day assay. The plate was sealed with Parafilm and incubated at 25 °C. Live and dead worms were manually counted every 24 h under a stereomicroscope Motic SMZ-161.

#### 2.6.2 Locomotor activity

The worm Adult Activity Test (wAAT) methodology was carried out, with some modifications (Hunt et al., 2023). wAAT protocol uses a WMicroTracker™ (Phylumtech, Argentina), an infrared beam interruption detection device from InVivo Biosystems (Simonetta and Golombek, 2007). Briefly, a total of 60–80 L4-stage nematodes were transferred to each well of a 96-well plate containing S medium for 60 min at 25 °C. Baseline motility was recorded for 30 min and normalized to 100% for each well. Subsequently, CFS was added at final concentrations of 25, 50, and 75% (v/v in S medium). The plate was sealed with Parafilm and monitored for 120 min at 25 °C. A second motility reading was taken after 24 h of incubation. S medium and MRS broth were included as negative and vehicle controls, respectively.

#### 2.6.3 Brood size

As the reproductive system of *C. elegans* is particularly susceptible to chemical toxicity, the impact of CFS exposure on nematode fertility was evaluated (Yang et al., 2015). L4-stage N2 nematodes (10 per well) were transferred to a 96-well plate containing 25, 50, or 75% CFS, along with heat-killed *E. coli* OP50 (A_600nm_ = 1). The plate was sealed with Parafilm and incubated at 25 °C for 72 h. To quantify progeny, the entire well volume was transferred to a 1.5 mL conical tube, followed by two washes with M9 buffer. Eggs and larvae were manually counted under a Motic SMZ-161 stereomicroscope, and the average progeny per treatment was calculated and compared to the untreated control. The percentage of offspring’s number (brood size) was plotted as a function of CFS concentrations.

### 2.7 *In vivo* antimicrobial activity of CFS in *Caenorhabditis elegans*

The protective effect of the CFS from *L. paraplantarum* CRL 1905 was further investigated against infections caused by clinical isolates of *S. aureus* and *P. aeruginosa,* using *C. elegans* as host model. Three approaches were used: (i) pre-treatment, where nematodes were in contact with the CFS for 24 h before infection; (ii) co-exposure, where CFS and bacteria were added simultaneously, and (iii) post-infection treatment, where nematodes were treated with CFS after infection with the pathogenic isolates. FUdR was added at a final concentration of 50 μM in liquid or solid media before solidification to prevent progeny. Nematode survival was assessed daily until all animals died and compared to the appropriate controls.

- Pre-treatment. Synchronized L4 *C. elegans* (*n* = ~100/treatment) were added to conical tubes of 1.5 mL, and incubated with CFS or MRS at 12.5, 25, or 50% in S medium for 24 h at 25 °C. Then, nematodes were washed twice with S medium, and ~20 animals per treatment (three replicates per condition, *n* = 60) were added to Tryptic Soy Agar (TSA) or modified NGM (0.35% peptone) plates seeded with *S. aureus* and *P. aeruginosa*, respectively (Powell and Ausubel, 2008). Bacteria were grown ON in liquid medium from a single colony, ~10^7^ CFU/mL were then inoculated onto TSA/NGM plates and incubated at 37 °C for 24 h. Plates with nematodes were incubated at 25 °C, and nematode survival was monitored daily under a Motic SMZ-161 stereomicroscope until all animals died.
- Co-exposure. Synchronized L4 *C. elegans* (15–20 per well) were added to a 96-well plate containing CFS or MRS at 12.5, 25, or 50% in S medium (eight replicates per treatment, *n* = 160), along with *S. aureus* or *P. aeruginosa* at ~10^7^ CFU/mL. Plates were incubated at 25 °C, and nematode survival was monitored daily under a Motic SMZ-161 stereomicroscope until all animals died.
- Post-infection treatment. Synchronized L4 *C. elegans* were infected for 24 h at 25 °C with *S. aureus* or *P. aeruginosa* seeded on TSA or modified NGM (0.35% peptone), respectively. Infection plates were prepared as described in (i). After infections, nematodes were washed three times with M9 buffer containing kanamycin (10 μg/mL) and streptomycin (100 μg/mL). Forty nematodes per well were then transferred to a 24-well plate containing CFS or MRS at 12.5, 25, or 50% in S medium, with heat-killed *E. coli* OP50 used as a food source (three replicates per treatment, *n* = 120). Plates were incubated at 25 °C, and nematode survival was monitored daily under a Motic SMZ-161 stereomicroscope until all animals died.

### 2.8 Statistical analysis

Data from *in vitro* and *in vivo* assays were analyzed using One-way ANOVA followed by Dunnett’s multiple comparisons test. In the infection assays, survival curves were generated using the Kaplan–Meier method, and comparisons were performed with the log-rank test, which appropriately incorporates the actual sample size of each group in the estimation and statistical comparison. Each condition included three independent biological replicates performed on different days with newly synchronized populations; technical replicates (wells or plates) are indicated for each assay. Statistical comparisons were performed using the Software GraphPad Prism version 8.0 for Windows (MA, USA). Differences at *p*-value < 0.05 were considered significant.

## 3 Results

### 3.1 Lactic acid bacteria exhibit a broad antimicrobial activity against different UP isolates

**Figure 1:**
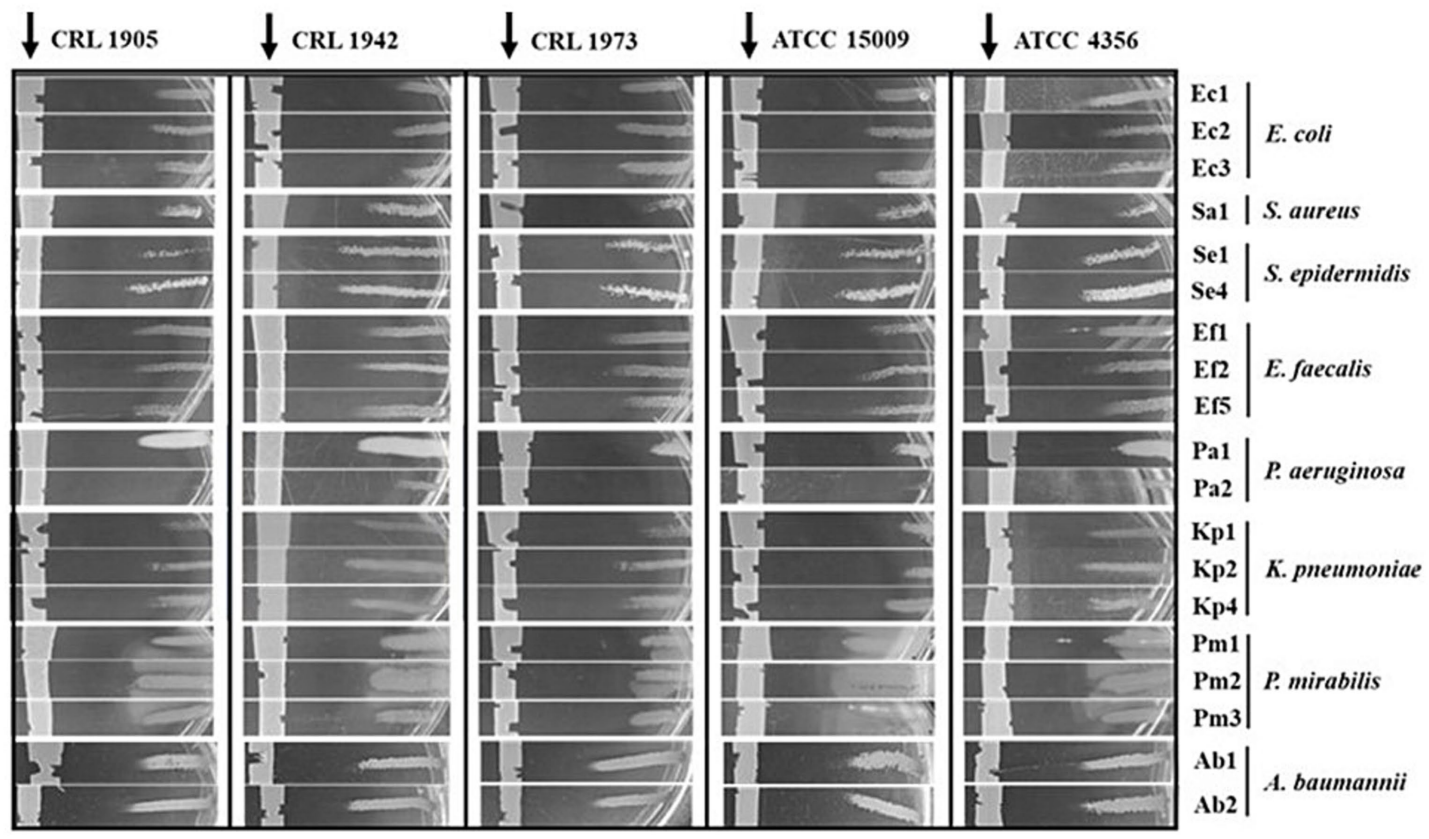
Antimicrobial activity assessed by the cross-streak assay. 20 μL of each LAB strain were streaked in the central area of BHI agar plates (arrows) and incubated for 24 h at 37 °C. Subsequently, perpendicular cross-streaks of the indicated UP were performed and incubated under the same conditions. Growth inhibition in the proximity of the central streak was considered a positive result. Images represent results from three independent experiments.

Five lactobacilli species, isolated from different sources, were tested for their potential antimicrobial activity: *L. paraplantarum* CRL 1905, *L. brevis* CRL 1942, *L. fermentum* CRL 973, *L. helveticus* ATCC 15009 and *L. acidophilus* ATCC 4356. Through a cross-streak assay, a preliminary screening was carried out against 19 UP clinical isolates, belonging to various genera and species (Table 1). As observed in Figure 1, all lactobacilli exhibited antibacterial activity against the tested pathogens, as evidenced by the UP-growth inhibition observed in the vicinity of the central streak.

**Figure 2:**
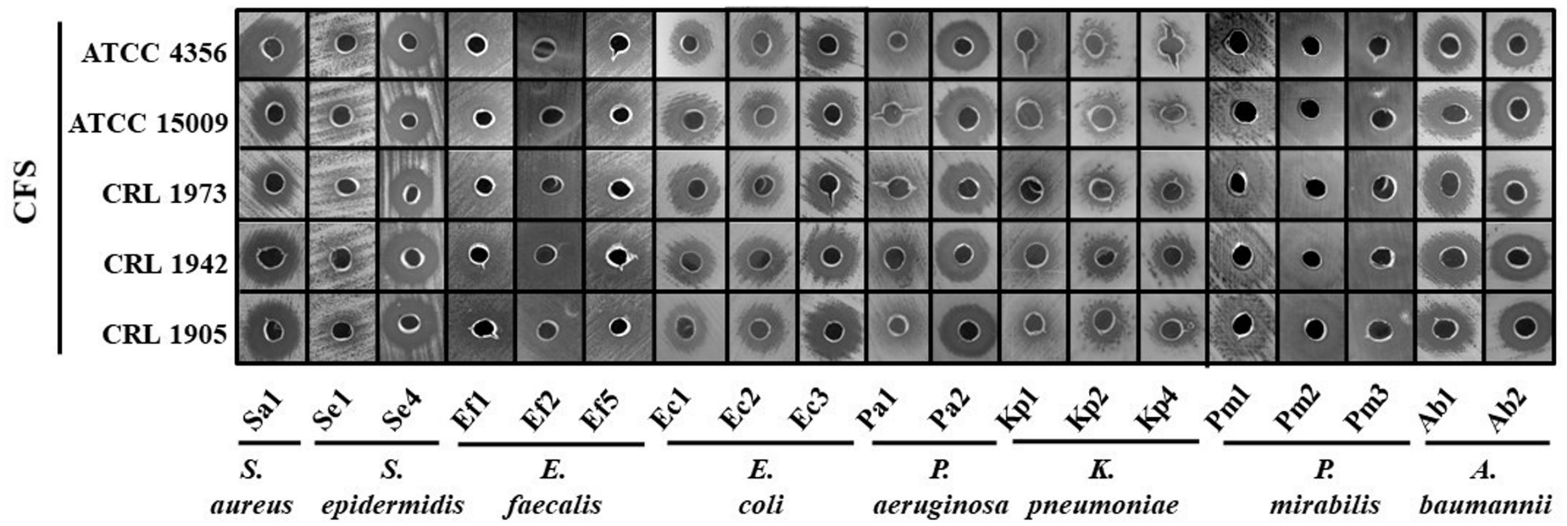
Antimicrobial activity assessed by the agar diffusion assay. CFSs from the indicated LAB strains were loaded into wells on MH agar plates seeded with the mentioned UP isolates. Then, plates were incubated at 37 °C for 24 h, and antimicrobial activity was evidenced as clear zones of growth inhibition around the wells. Images represent results from three independent experiments.

To determine whether the observed antimicrobial activity was due to direct cell–cell contact or secreted metabolites, 24 h CFS from each lactobacilli strain was tested against the UP. As shown in Figure 2, all CFSs exhibited antibacterial activity against all clinical isolates, with variable degrees. A marked inhibition was observed against *S. aureus* Sa1, *S. epidermidis* Se4, *E. coli* Ec1, *P. aeruginosa* Pa2, *P. mirabilis* Pm1, Pm2, and Pm3, *K. pneumoniae* Kp1 and Kp2, and *A. baumannii* Ab1 and Ab2, with halos ranging from 13 to 15.5 mm. In contrast, *S. epidermidis* Se1 and *K. pneumoniae* Kp4 exhibited reduced susceptibility with inhibition halos ≤ 10 mm. Based on these results, *P. aeruginosa* Pa2 (Gram-negative) and *S. aureus* Sa1 (Gram-positive) were selected as representative strains for further studies.

MIC values of the different CFSs were determined by the microdilution method and are presented in Table 2. Interestingly, CFSs from all lactobacilli strains showed a complete growth inhibition of both pathogens at a concentration of ≥ 25%. Regarding MBC, 25 and 50% concentrations were required to eliminate 99.9% of Sa1 and Pa2 cells, respectively.

**Table 2:** MIC and MBC of the CFSs against the selected UP.

CFS	*S. aureus* Sa1		*P. aeruginosa* Pa2	
	MIC	MBC	MIC	MBC
CRL 1905	25	≥ 25	25	≥ 50
CRL 1942	25	≥ 25	25	≥ 50
CRL 973	25	≥ 25	25	≥ 50
ATCC 15009	25	≥ 25	25	≥ 50
ATCC 4356	25	≥ 25	25	≥ 50

MIC and MBC are CFS concentrations in % (v/v).

### 3.2 CFSs exert antimicrobial activity through combined factors

In order to characterize the nature of the inhibitory substances secreted by each LAB strain, CFSs were subjected to different treatments and their antimicrobial activity was subsequently tested (Table 3). The ability of CFSs to inhibit the selected UP growth was completely lost only after pH neutralization (pH 6.5), inferring an acidic nature of the metabolite/s involved. Sterile MRS adjusted to pH 4 (the natural pH of the CFSs) did not show antimicrobial activity. Similarly, treatment with lactic acid -the major organic acid produced by these strains- did not affect the growth of the tested UP. These results indicate that neither acidification alone nor lactic acid at the tested concentration accounted for the antimicrobial activity observed in the CFSs. In addition, the activity of CFSs against Sa1 was partially affected by high temperatures and protease treatment, suggesting that part of their antagonistic capacity was due to the presence of proteinaceous molecules.

**Table 3:** Effect of pH, heat treatment and enzymes on CFSs antimicrobial activities against the selected UP.

CFS	Inhibition zone diameter (mm)				
	Untreated	*N*	Ø	PK	Tryp
CRL 1905					
Sa1	13.0 ± 2.1	nd	10.5 ± 0.7	11.0 ± 1.4	9.0 ± 1.2
Pa2	12.0 ± 1.4	nd	11.0 ± 2.1	11.0 ± 0.7	12.0 ± 1.4
CRL 1942					
Sa1	12.0 ± 0.7	nd	9.5 ± 0.7	9.5 ± 2.1	8.0 ± 1.2
Pa2	10.0 ± 1.4	nd	10.0 ± 2.1	9.5 ± 0.7	10.5 ± 0.7
CRL 1973					
Sa1	12.0 ± 2.8	nd	9.5 ± 0.7	9.5 ± 2.1	8.0 ± 1.2
Pa2	10.0 ± 2.1	nd	9.0 ± 2.1	9.0 ± 0.7	10.0 ± 1.4
ATCC15009					
Sa1	12.0 ± 0.7	nd	9.5 ± 0.7	10.5 ± 2.1	10.0 ± 0.7
Pa2	12.0 ± 1.4	nd	12.0 ± 1.4	10.0 ± 1.4	12.0 ± 2.1
ATCC4356					
Sa1	14.5 ± 3.5	nd	10.0 ± 1.4	9.5 ± 0.7	9.0 ± 1.2
Pa2	12.0 ± 1.4	nd	12.5 ± 2.1	11.0 ± 0.7	10.5 ± 2.1

*N*, neutralization; Ø, heat; PK, proteinase K; Tryp, trypsin.

nd, not detected.

Data are presented as the mean value ± standard deviation of three independent measurements.

### 3.3 Lactobacilli-derived CFSs inhibit formation and disrupt mature UP biofilms

**Figure 3:**
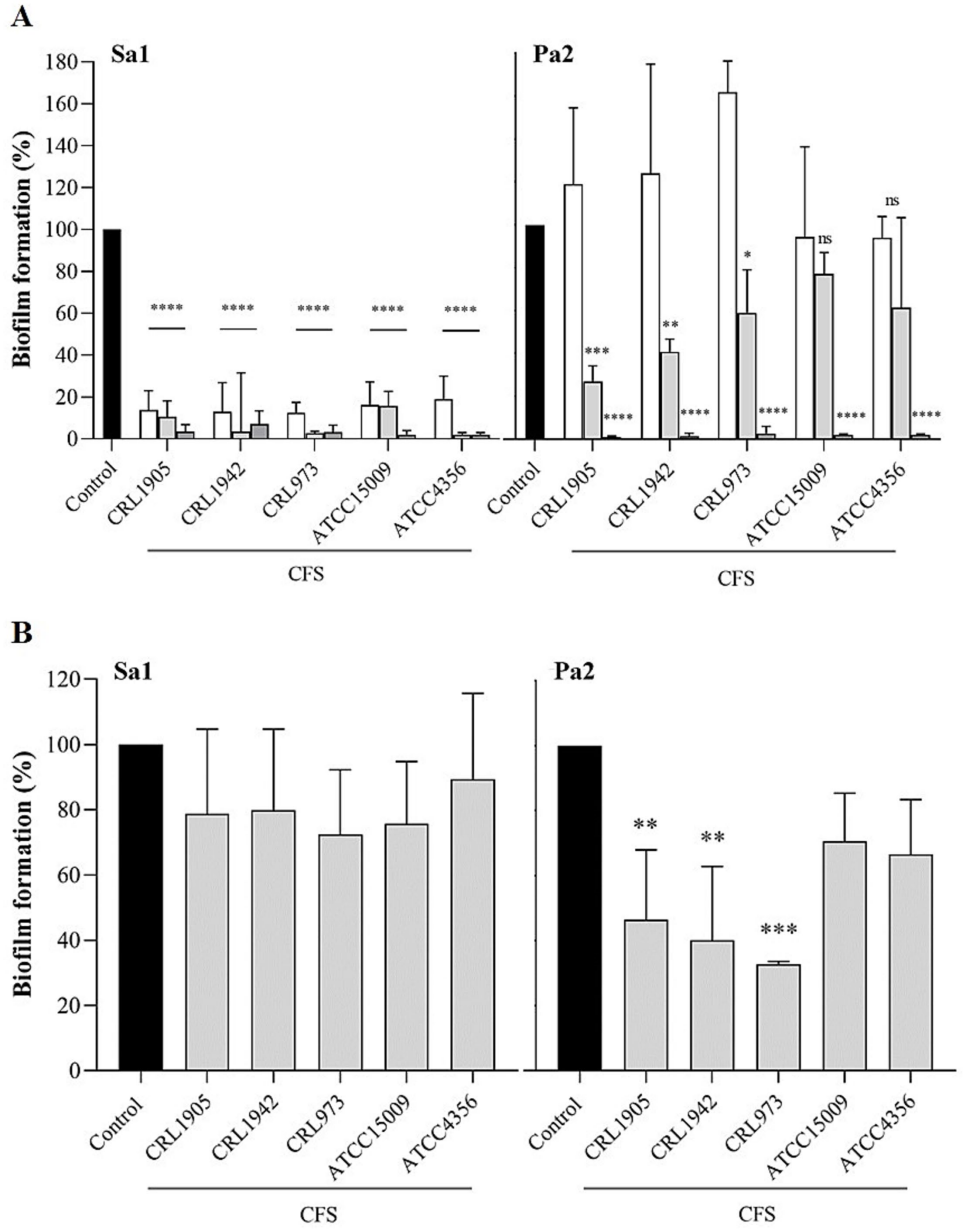
Inhibition and eradication of UP biofilms. Inhibition of biofilm formation of *S. aureus* Sa1 (left panel) and *P. aeruginosa* Pa2 (right panel) **(A)**. Cells were incubated with different concentrations of LAB CFSs for 48 h at 37 °C, and biofilm biomass was quantified after staining with 0.1% crystal violet. CFSs: 6.25% (white), 12.5% (light grey), and 25% (dark grey). Eradication of preformed biofilms **(B)**. Established 48-h biofilms of *S. aureus* Sa1 (left panel) and *P. aeruginosa* Pa2 (right panel) were incubated with 10x CFSs from different LAB strains at 37 °C for 24 h, followed by staining with 0.1% crystal violet. For each panel, data represent the mean ± SD of three independent experiments. Statistical significance is indicated as ns (not significant), *p ≤ 0.05, **p ≤ 0.01, ***p ≤ 0.001, ****p ≤ 0.0001. Exact *p*-values for Pa2 biofilm inhibition (shown in order of increasing CFS concentration) were: CRL1905 (*p* = 0.5405, *p* = 0.0002, *p* < 0.0001); CRL1942 (*p* = 0.3523, *p* = 0.0028, *p* < 0.0001); CRL973 (*p* = 0.0018, *p* = 0.0470, *p* < 0.0001); ATCC15009 (*p* = 0.9733, *p* = 0.3826, *p* < 0.0001); and ATCC4356 (*p* = 0.9662, *p* = 0.0652, *p* < 0.0001). For Sa1 biofilm inhibition, all comparisons showed **** significance (*p* < 0.0001). For eradication assays, adjusted *p*-values for Pa2 strain (in order of strain appearance) were 0.0053, 0.0023, 0.0009, 0.1409, and 0.0838, whereas all comparisons for Sa1 strain were ns.

Pathogen biofilm formation is one of the mechanisms responsible for the prolonged bacterial persistence in the genitourinary tract (Vuotto et al., 2017). Cells in biofilm are up to 1,000 times more resistant to conventional antibiotic treatment than those in the planktonic state, leading to structures that are extremely difficult to eradicate. Here, the potential use of lactobacilli CFSs as an alternative to prevent and/or treat biofilms caused by *S. aureus* Sa1 and *P. aeruginosa* Pa2 was assessed. Both strains were strong biofilm producers when grown for 48 h in BHI medium (Figure 3). All CFSs were able to inhibit UP biofilm formation in a dose-dependent manner, requiring a concentration of 12.5% (1/2 MIC) to completely inhibit biofilm formation of both pathogens (Figure 3). It is worth mentioning that even a CFS concentration of 6.25% (1/4 MIC) led to a ~ 70% reduction in *S. aureus* Sa1 biofilm formation. In addition, the CFSs’ ability to eradicate preformed biofilms of both UP was evaluated by treating 48-h mature biofilms with 10-fold concentrated CFSs for 24 h, followed by quantification of residual biofilm biomass. Results showed that treatment with any supernatant was unable to disrupt the Sa1 preformed biofilm (Figure 3). However, Pa2 mature biofilm was significantly dispersed by CFSs of *L. paraplantarum* CRL 1905, *L. brevis* CRL 1942, and *L. fermentum* CRL 973 strains. *L. helveticus* ATCC 15009 and *L. acidophilus* ATTC 4356 CFSs did not reduce pre-formed biofilm (Figure 3). Overall, while all CFSs effectively inhibited biofilm formation in both pathogens, their ability to affect established biofilms differed between species. Mature *P. aeruginosa* biofilms were more susceptible to treatments, whereas preformed *S. aureus* biofilms showed limited susceptibility to eradication. This dual activity is critical to effectively manage persistent infections and improve the efficacy of antimicrobial therapies.

### 3.4 CFS from *Lactiplantibacillus paraplantarum* CRL 1905 exhibits dose-dependent toxicity in *Caenorhabditis elegans*

**Figure 4:**
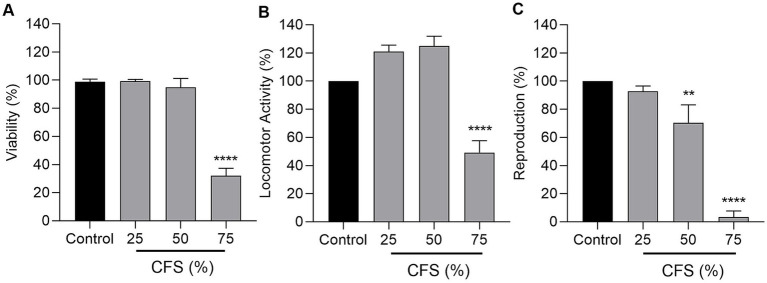
*Caenorhabditis elegans* biosafety. *C. elegans* N2 viability was determined after 24 h exposure to CFS from *L. paraplantarum* CRL 1905 **(A)**. *C. elegans* N2 locomotor activity (%) was measured after 24 h of CFS from *L. paraplantarum* CRL 1905 exposure using the Worm Motility Tracker (WMT). Data represent the average locomotor activity of nematode populations from three replicate wells over time **(B)**. *C. elegans* N2 reproduction (%) in the presence of CFS from *L. paraplantarum* CRL 1905 was evaluated at 24 h by counting eggs and larvae **(C)**. Controls included worms treated with MRS and S medium, and was considered as 100% locomotor activity or expressed as 100% reproduction. Data are the result of three independent experiments and are expressed as mean ± SD with *p* ≤ 0.05. ***p ≤ 0.001, ****p ≤ 0.0001 (*n*: 180–240 worms per treatment).

Evaluating the safety of CFS is a critical step before considering its therapeutic or preventive application. The *in vivo* model *C. elegans* offers a valuable and ethically accepted system to assess potential toxicological effects on host viability, reproduction, and physiology (Powell and Ausubel, 2008; Wani et al., 2020; Kurz and Ewbank, 2000). Based on its robust antimicrobial and antibiofilm activity, CFS from *L. paraplantarum* CRL 1905 was selected for further studies. First, to evaluate the acute and chronic toxic potential of this postbiotic, nematode viability was monitored every 24 h throughout a 10-day exposure period to increasing CFS concentrations (0, 25, 50, and 75%). During the treatment, 25 and 50% CFS did not affect viability in comparison with the control group (without CFS). In contrast, exposure to 75% CFS induced a marked viability reduction, which dropped to 30 ± 4% within 24 h (*p <* 0.0001) (Figure 4), indicating a strong acute toxic effect. By day 3, all nematodes from 75% treatment were dead.

When evaluating the locomotor activity, a similar response was observed with 75% CFS, which caused a paralysis of more than half of the nematodes at 24 h (*p <* 0.0001) (Figure 4). The parallel decline in survival and motility suggests that impaired movement is closely linked to overall viability loss. Notably, locomotor inhibition appears as an earlier and more sensitive indicator of toxicity, whereas viability reflects the cumulative outcome of such physiological damage.

Finally, reproduction was evaluated as a key endpoint of chronic toxicity in *C. elegans*, since brood size is susceptible to stress and metabolic disruption, and reflects the overall fitness and reproductive success of the organism. Therefore, brood size was quantified 72 h after exposure to different CFS concentrations. The egg-laying capacity of *C. elegans* was unaffected when exposed to 25% CFS, whereas a concentration of 50% caused a decrease of 30 ± 10%, when compared to controls (*p =* 0.0022) (Figure 4). Taken together, our results demonstrate that CFS is safe at concentrations up to 25%, as no adverse effects were observed on the general physiology of *C. elegans*.

### 3.5 CFS from *Lactiplantibacillus paraplantarum* CRL 1905 enhances survival of *Caenorhabditis elegans* infected with *Staphylococcus aureus* and *Pseudomonas aeruginosa*

*C. elegans* is a well-established infection model system to study bacterial pathogenesis, as it shares common virulence and host defense mechanisms with humans (Darby, 2005; Irazoqui et al., 2010). Since no relevant adverse effects were observed in *C. elegans* viability at concentrations up to 50% of the CFS from *L. paraplantarum* CRL 1905, this range was used to evaluate its impact on the infection model with *S. aureus* and *P. aeruginosa*.

**Figure 5:**
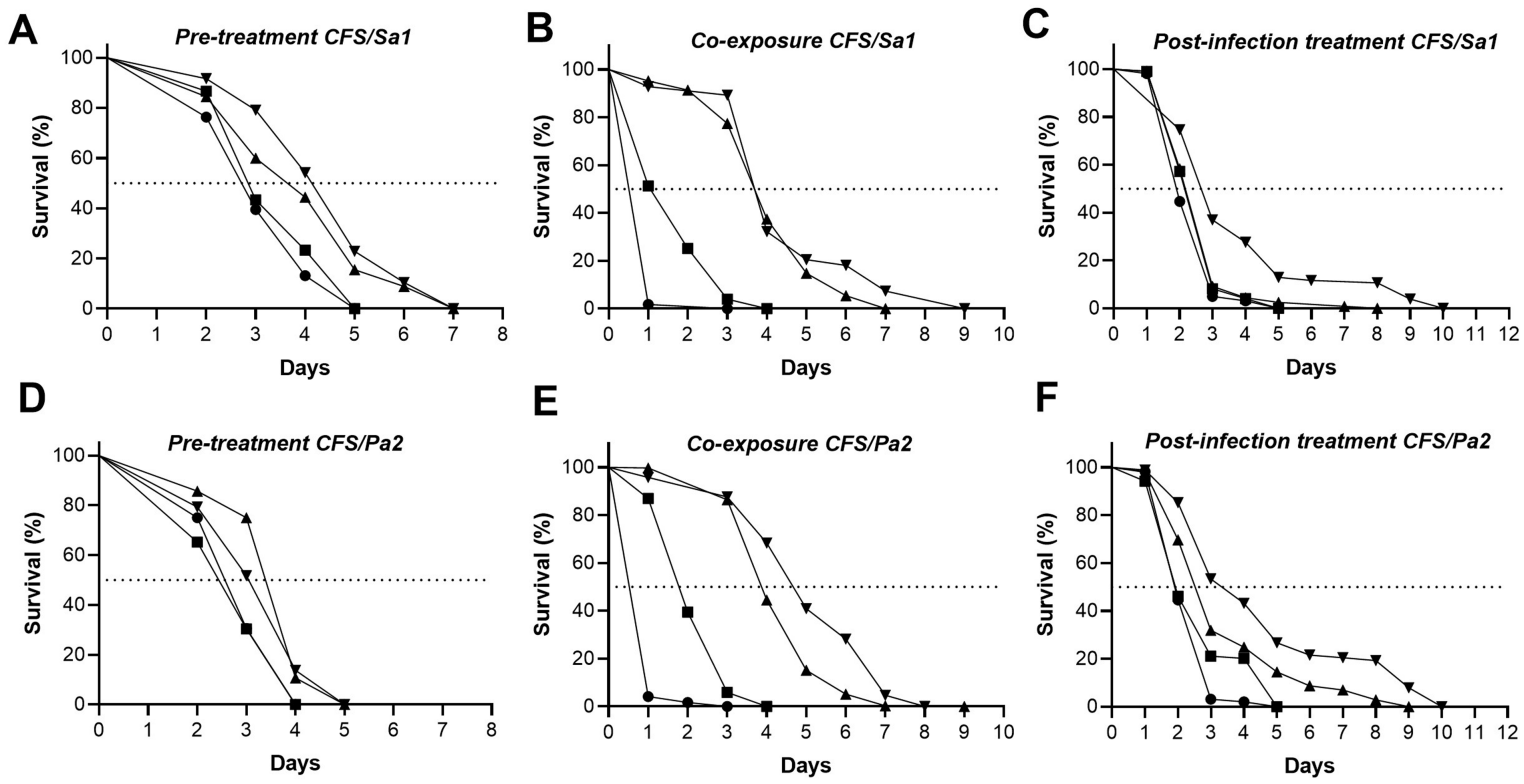
Effect of CFS from *L. paraplantarum* CRL 1905 on *C. elegans* survival during *S. aureus* Sa1 **(A–C)** and *P. aeruginosa* Pa2 **(D–F)** infection. Worms were treated with different concentrations of CFS before **(A,D)**, during **(B,E)**, or after infection **(C,F)**. 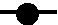 without CFS, 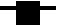 CFS 12.5%, 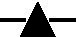 CFS 25%, and 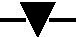 CFS 50%. Survival was monitored over time, and curves were generated using the Kaplan–Meier method. Data are the result of three independent experiments. Statistical comparison was performed using the log-rank test. Differences were considered significant when *p* ≤ 0.05. **** p ≤ 0.0001.

The treatment strategies employed to assess the impact of CFS on *C. elegans* revealed a concentration-dependent improvement in the survival of the nematodes. When worms were pretreated with CFS for 24 h prior to infection with *S. aureus* or *P. aeruginosa,* an improvement in median survival was observed. Against *S. aureus*, survival increased from 2.5 days in the control group to 3 and 4 days with 25 and 50% CFS, respectively (*p =* 0.0016 and *p <* 0.0001). Similarly, against *P. aeruginosa,* survival was extended from 3 days in the control to 4 days at both CFS concentrations (*p =* 0.0005 for 25% and *p* = 0.0392 for 50%) (Figure 5). Simultaneous exposure of nematodes to CFS and pathogens markedly improved median survival, increasing from 1 day in the control to 4 days with 25 and 50% CFS against *S. aureus* (*p <* 0.0001), and up to 5 days with 50% CFS against *P. aeruginosa* (*p <* 0.0001) (Figure 5). Finally, when worms were treated with CFS after the infection, median survival increased from 2 to 3 days with 50% CFS against *S. aureus* (*p <* 0.0001), and up to 4 days at the same concentration against *P. aeruginosa* infection (*p <* 0.0001) (Figure 5). As shown in Figure 5, treatments with 12.5% CFS had minimal impact on *C. elegans* survival, resulting in curves comparable to the untreated control. These results demonstrate that CFS exhibited therapeutic potential even after infection was established in the nematode model system.

## 4 Discussion

UTIs are one of the most common and frequently recurring bacterial infections in people of all ages, becoming a worldwide therapeutic problem. In recent years, there has been a significant increase in the emergence of resistant pathogens due to long-term use of ATB. This problem requires the development of alternative strategies to prevent and treat these infections. Among the most prominent approaches, the use of probiotics, postbiotics, and immunostimulants is of special interest due to their antagonistic capacities (El-Mokhtar et al., 2020; Kiousi et al., 2023). Probiotics have become a hot spot in research due to their GRAS status, nutritional and health value, and antimicrobial activity. Here, all tested lactobacilli strains exhibited broad inhibitory capacity against both Gram-positive and Gram-negative UP, inferring a general antimicrobial mechanism. Most of the tested pathogens, belonging to the ESKAPE group (acronym comprising the scientific names of *E. faecium, S. aureus, K. pneumoniae, A. baumannii, P. aeruginosa,* and *Enterobacter spp*), have acquired resistance to a wide spectrum of ATB, and thereby representing a great challenge for their eradication (Farizano et al., 2025; Pendleton et al., 2013; Mulani et al., 2019). Similarly, Rastogi et al. (2021) reported that several LAB strains exhibited inhibitory activity against all ESKAPE pathogens, with a variable degree of antagonism.

Since the use of live microorganisms persists as a debated issue due to safety concerns, especially for immunocompromised individuals (Kullar et al., 2023), non-viable microorganisms, their fragments, or products released in CFS, also referred to as “postbiotics,” represent an interesting advantage as biotherapeutics (Piqué et al., 2019; Calvanese et al., 2025). Our results showed that all CFSs retained the broad inhibitory activity exhibited by the whole cells against the tested UP, highlighting their potential as a promising strategy to control multiple pathogens. The use of metabolite-based products offers several advantages over live bacterial cells, including improved stability and reduced sensitivity to environmental factors such as temperature, oxygen exposure, and prolonged storage.

MBC values were either comparable to or twice as high as MIC values for the Sa1 and Pa2 strains, respectively. Levison (2004) reported that therapeutic drugs are bactericidal when the MBC is usually the same as the MIC, and generally, not more than four-fold higher, indicating that all CFSs exhibited a bactericidal effect against both pathogens. This bactericidal activity is particularly relevant in the context of treating infections caused by opportunistic or antibiotic-resistant pathogens, where the complete elimination of bacterial cells is desirable to prevent persistence or recurrence.

Our results indicate that the inhibitory effect of CFSs may be related to organic acids or bacteriocins that require low pH to remain stable or active. However, since acidified MRS did not exert inhibitory activity, the acidic environment per se was discarded as responsible for bacterial inhibition. Several studies have demonstrated that many bacteriocins produced by *Lactobacillus* species, such as plantaricins or paraplantaricins, are often pH-dependent, exhibiting maximal activity under acidic conditions (De Vuyst and Leroy, 2007; Wala’a and Nibras, 2013). Additionally, organic acids, such as lactic and acetic acid, not only reduce environmental pH but can also diffuse across bacterial membranes in their undissociated form, dissociate intracellularly, and disrupt key metabolic processes- a mechanism that is strongly pH-dependent (Alakomi et al., 2000).

Volentini et al. (2023) demonstrated that CFS from *L. paraplantarum* CRL 1905 exhibited antimicrobial activity against fungi, underscoring its potential as a broad-spectrum producer of bioactive metabolites. This strain produced lactic, acetic, propionic, and phenyllactic acids, compounds with well-documented antimicrobial activity. Organic acids tend to interact with bacterial membranes, inducing morphological and physiological alterations that lead to membrane damage and leakage of cellular contents (Ji et al., 2023). Among them, phenyllactic acid, a typical metabolite derived from LAB, has demonstrated a broad-spectrum antimicrobial activity (Siedler et al., 2019). This compound inhibits the growth of both Gram-positive and Gram-negative bacteria by disrupting membrane integrity or by interfering with bacterial DNA replication through intercalation (Ning et al., 2021). Although phenyllactic acid alone can exert antimicrobial effects, several studies suggest that it is unlikely that a single organic acid could be solely responsible for the observed activity. Instead, it is more plausible that a combination of organic acids exerts additive or synergistic effects that enhance bacterial growth inhibition (Ji et al., 2023; Abdul-Rahim et al., 2021). On the other hand, the partial loss of CFS antimicrobial activity by protease or heat treatment suggested the antibacterial action of proteinaceous molecules in the supernatant. Taken together, our findings indicate that the antimicrobial activity of the CFS may be multifactorial, as a result of the combined and potentially synergistic action of organic acids and bacteriocins.

Postbiotics are being increasingly investigated for their potential antibiofilm properties. Our study demonstrated the potential efficacy of CFSs derived from all LAB strains to inhibit *S. aureus* biofilm formation, at concentrations below MIC, indicating that the observed result was not due to the pathogen’s death. Melo et al. (2016) demonstrated that the supernatant from *L. fermentum* TCUESC0 also reduced biofilm formation of *S. aureus* CCMB262 at subinhibitory concentrations. CFS components may interfere with biofilm formation by disrupting microbial co-aggregation, or by impairing *S. aureus* adherence to solid surfaces. On the other hand, the lower efficacy of the CFS in inhibiting *P. aeruginosa* biofilms may be linked to the intrinsic resistance of this bacterium to various ATB (Alexandre et al., 2014; El-Mokhtar et al., 2020). This bacterium exhibits limited permeability of its outer membrane and expresses efflux pumps that can expel different antimicrobial agents from the cell, which would complicate bacterial eradication and perpetuate persistent infections (Breidenstein et al., 2011; Alexandre et al., 2014; Pang et al., 2019; El-Mokhtar et al., 2020). Furthermore, several virulence factors, such as elastolytic activity and the capacity to form dense, structured biofilms, may also limit the effectiveness of the CFS, enabling *P. aeruginosa* to partially withstand its inhibitory effects.

Interestingly, while *S. aureus* biofilm formation was more susceptible to CFS inhibition in early stages, the preformed biofilm of this species was largely resistant to disruption. In contrast, *P. aeruginosa* biofilm, although initially less affected during formation, appeared more vulnerable to disruption once established. This apparent paradox may reflect differences in biofilm structure, composition, and dynamics between the two species. *S. aureus* is known to enter a persistent, metabolically inactive state in mature biofilms, which can confer increased tolerance to antimicrobials (Otto, 2008; Conlon, 2014). Moreover, the thick peptidoglycan layer may limit the access or diffusion of certain CFS components once the biofilm has matured. In contrast, *P. aeruginosa* biofilms, although structurally complex, are highly dynamic and metabolically active, particularly in deeper layers, which could make them more susceptible to the action of certain CFS components, such as organic acids or biosurfactants capable of disrupting membrane integrity or interfering with matrix cohesion (Davares et al., 2022; Nataraj et al., 2021; Drumond et al., 2023). Understanding these mechanisms may be critical for optimizing the use of LAB-derived postbiotics as anti-biofilm agents.

Following the *in vitro* assays, the toxicity and antimicrobial activity of CFS were further evaluated *in vivo* using *C. elegans*, a well-established model that provides insights from a whole animal with metabolically active digestive, reproductive, endocrine, sensory, and neuromuscular systems (Hunt, 2017). This nematode is increasingly recognized as a reliable and cost-effective alternative for evaluating probiotic- and postbiotic-based therapeutic strategies (Gravato-Nobre and Hodgkin, 2005). In our study, exposure to 25 and 50% CFS did not affect viability or locomotor activity, indicating that these concentrations are non-toxic; however, 50% CFS reduced brood size, demonstrating a subtle impact on reproductive capacity. Viability measures overall survival and reflects lethal effects, whereas locomotor activity detects sublethal impairments in neuromuscular function or general physiological status, providing an early indicator of toxicity. Reproduction reveals effects on fertility and developmental processes that may occur even when survival appears normal (Dhawan et al., 1999).

*C. elegans* is also an interesting model for studying host–pathogen interactions and innate defense mechanisms, given its evolutionary conservation with higher organisms and its experimental tractability (Darby, 2005; Irazoqui et al., 2010). Our study revealed that CFS from *L. paraplantarum* CRL 1905 improved *C. elegans* survival when administered either before, during, or after infection with *S. aureus* or *P. aeruginosa*. These protective effects could be explained by multiple mechanisms, non–mutually exclusive processes, including direct antimicrobial or anti-virulence effects on the pathogens or host-mediated responses. Although specific immune pathways were not examined, a previous work have shown that probiotic-derived metabolites can modulate innate immunity in *C. elegans* (e.g., p38 MAPK), providing a plausible explanation for the enhanced survival observed in our assays (Dinić et al., 2021). Consistent with our findings, differences in nematode survival between infections with *S. aureus* and *P. aeruginosa* likely reflect the distinct infection strategies and pathogenic mechanisms of each microorganism. *P. aeruginosa* can cause death either through slow killing, mediated by intestinal colonization and chronic virulence factor production, or through fast killing driven by diffusible toxins in liquid or nutrient-limited media (Tan et al., 1999; Irazoqui et al., 2010; Anderson et al., 2018). In contrast, *S. aureus* typically kills via intestinal accumulation, accompanied by progressive epithelial damage (Sifri et al., 2003; Irazoqui et al., 2010).

Across all treatments, survival increased in a concentration-dependent manner, with the strongest effects observed during co-exposure with the highest concentration. Our findings indicate that although 50% CFS provided the greatest protection, it also impaired reproduction. Meanwhile, 25% CFS preserved viability, motility, and reproductive capacity, and still conferred a significant protection, suggesting that this concentration may represent the optimal balance between efficacy and safety. Importantly, the toxicity observed at 75% CFS corresponds to the highest concentration evaluated to defined the safety threshold within the *C. elegans* model rather than a therapeutically relevant exposure level. Protective effects against both pathogens were consistently observed at substantially lower concentrations (25–50%), indicating the presence of a functional therapeutic window. In practical applications, postbiotics are typically present at diluted concentrations due to physiological fluids or formulation constraints (Aguilar-Toalá et al., 2018; Salminen et al., 2021). Therefore, the protective activity observed at 25% CFS likely falls within a range compatible with safe host exposure. Together, these considerations support the translational relevance of the protective effects observed in this model.

Interestingly, while many LAB-derived postbiotics display selective antimicrobial effects depending on the bacterial type, the CFS from *L. paraplantarum* CRL 1905 conferred protection against both Gram-positive and Gram-negative pathogens in *C. elegans*. This broad-spectrum effect contrasts with findings from other well-studied strains, such as *L. acidophilus* NCFM, which exhibited pronounced activity against Gram-positive bacteria but displayed a minimal inhibitory effect on infections with *P. aeruginosa* or *S. enterica* (Kim and Mylonakis, 2012). The broad-spectrum efficacy of CFS from *L. paraplantarum* CRL 1905 highlights its value as a versatile postbiotic candidate, with promising potential as an adjunctive strategy to counteract antibiotic-resistant infections.

## 5 Conclusion

In conclusion, this study demonstrates that LAB-derived postbiotics exert broad antimicrobial and antibiofilm activities against clinically relevant UP and can enhance host survival in an invertebrate infection model. Our findings highlight the potential of LAB supernatants as an alternative approach to ATB in the treatment of UTI caused by both Gram-positive and Gram-negative pathogens, and offer new perspectives for addressing the rising challenges of hospital-acquired infections and bacterial resistance. Future studies, including metabolomic analyses and validation in mammalian models, will be essential to better elucidate mechanisms of action and further evaluate their therapeutic potential.

## Data Availability

The original contributions presented in the study are included in the article/supplementary material, further inquiries can be directed to the corresponding author.
